# Effects of Extracorporeal Shockwave Therapy on Functional Recovery and Circulating miR-375 and miR-382-5p after Subacute and Chronic Spinal Cord Contusion Injury in Rats

**DOI:** 10.3390/biomedicines10071630

**Published:** 2022-07-07

**Authors:** Mohamed Ashmwe, Katja Posa, Alexander Rührnößl, Johannes Christoph Heinzel, Patrick Heimel, Michael Mock, Barbara Schädl, Claudia Keibl, Sebastien Couillard-Despres, Heinz Redl, Rainer Mittermayr, David Hercher

**Affiliations:** 1 The Research Center in Cooperation with AUVA, Ludwig Boltzmann Institute for Traumatology, 1200 Vienna, Austria; mohamed.ashmwe@trauma.lbg.ac.at (M.A.); katja.posa@gmail.com (K.P.); alexander.ruehrnoessl@trauma.lbg.ac.at (A.R.); patrick.heimel@trauma.lbg.ac.at (P.H.); michael.mock@trauma.lbg.ac.at (M.M.); barbara.schaedl@trauma.lbg.ac.at (B.S.); claudia.keibl@meduniwien.ac.at (C.K.); heinz.redl@trauma.lbg.ac.at (H.R.); rainer.mittermayr@trauma.lbg.ac.at (R.M.); 2 Austrian Cluster for Tissue Regeneration, 1200 Vienna, Austria; s.couillard-despres@pmu.ac.at; 3 Department of Hand, Plastic, Reconstructive and Burn Surgery, University of Tuebingen, 72074 Tübingen, Germany; jheinzel@bgu-tuebingen.de; 4 Core Facility Morphology, University Clinic of Dentistry, Medical University of Vienna, 1090 Vienna, Austria; 5 Institute of Experimental Neuroregeneration, Spinal Cord Injury & Tissue Regeneration Centre Salzburg (SCI-TreCS), Paracelsus Medical University, 5020 Salzburg, Austria; 6 Department Life Science Engineering, University of Applied Sciences Technikum Wien, 1200 Vienna, Austria

**Keywords:** spinal cord injury, neuroregeneration, extracorporeal shockwave therapy, microRNAs, contusion injury, motor function

## Abstract

Extracorporeal shockwave therapy (ESWT) can stimulate processes to promote regeneration, including cell proliferation and modulation of inflammation. Specific miRNA expression panels have been established to define correlations with regulatory targets within these pathways. This study aims to investigate the influence of low-energy ESWT—applied within the subacute and chronic phase of SCI (spinal cord injury) on recovery in a rat spinal cord contusion model. Outcomes were evaluated by gait analysis, µCT and histological analysis of spinal cords. A panel of serum-derived miRNAs after SCI and after ESWT was investigated to identify injury-, regeneration- and treatment-associated expression patterns. Rats receiving ESWT showed significant improvement in motor function in both a subacute and a chronic experimental setting. This effect was not reflected in changes in morphology, µCT-parameters or histological markers after ESWT. Expression analysis of various miRNAs, however, revealed changes after SCI and ESWT, with increased miR-375, indicating a neuroprotective effect, and decreased miR-382-5p potentially improving neuroplasticity via its regulatory involvement with BDNF. We were able to demonstrate a functional improvement of ESWT-treated animals after SCI in a subacute and chronic setting. Furthermore, the identification of miR-375 and miR-382-5p could potentially provide new targets for therapeutic intervention in future studies.

## 1. Introduction

Extracorporeal shockwave therapy (ESWT) has proven to be effective for various pathologies in different tissues [1,2,3,4,5,6]. Recently, experimental as well as clinical studies have shown the beneficial effects of ESWT in both the peripheral and the central nervous system [7,8,9,10,11,12,13,14,15]. Furthermore, basic research studies have enabled a better understanding of the underlying mechanisms of shockwave therapy in biological tissues. They include the release of various growth factors, enhanced cell proliferation and migration, recruitment of stem cells, influence of cell–cell communication, improvement of the local tissue perfusion by angiogenesis and the modulation of inflammation [11,12,13,14,16,17].

Shockwaves are defined as types of acoustic pressure waves that develop during sudden releases of energy with a specific time-to-pressure curve profile [18,19]. The translation of the exogenously applied acoustic pressure waves to biochemical signals, also known as mechanotransduction, effectively stimulates the body’s intrinsic repair and regeneration mechanisms.

Micro RNAs (miRNA) are a type of ribonucleic acid approximately 22 nucleotides long. miRNAs modulate gene expression at the mRNA level, by either promoting the degradation of the target mRNA or by inhibiting translation. Each miRNA can have multiple regulatory targets [20]. Furthermore, miRNAs circulate [21] and have systemic effects [22]. Blood plasma contains miRNA, which has previously been used as a biomarker for injuries to various tissues, including the brain [23], various types of cancer [24,25] and Alzheimer‘s disease [26]. Most miRNAs are conserved across species [27,28,29]. Therefore, information gathered from rats is expected to be translatable to humans. Specific miRNA expression panels have been established to correlate with apoptosis, inflammation, angiogenesis, oligo-dendrocyte development, axonal regeneration and remyelination after spinal cord injury (SCI) [30]. However, as a number of miRNAs are known to be up- and downregulated depending on the specific timepoint after SCI—such as miR-100, which is involved in apoptosis and inflammation [30]—recognition of dynamic changes in expression patterns presents a challenging task in investigating biological effects and correlations. Additional questions arise considering the highly complex manner in which miRNAs function within their cascades, such as with miR-375, which acts via p53 in a neuroprotective way [31], or miR-382, which is involved with microglial cells and the inflammatory response upon upregulation after SCI [32].

Mechanical stimulation of cells via shockwaves has recently been reported to increase exosome release [33]. These nanovesicles contained miRNAs, which induced a very potent angiogenic effect. We investigated a wide range of plasma-derived miRNAs present after spinal cord injury as well as after ESWT in order to identify any injury-, regeneration- and treatment-specific miRNA expression patterns. In addition to the evaluation of miRNA levels after SCI and ESWT, this study aimed to investigate the influence of low-energy ESWT on functional recovery in a rat contusion injury model within the sub-acute and chronic phase after a traumatic spinal cord injury, i.e., in the period in which secondary injury mechanisms take place.

## 2. Materials and Methods

### 2.1. Animals and Surgery

After ethical approval of the experimental protocol (TVA-Nr.: MA58/005956/2012/6) by the Animal Protocol Review Board of the City Government of Vienna, male Sprague-Dawley rats weighing 200–250 g were obtained from Janviers Labs (Le Genest-Saint-Isle, France). The animals were provided with ad libitum access to food and water and were housed under simulated daylight conditions with alternating 12-h light–dark cycles. Rats were familiarized with the new environment for a period of one week. One day before the operation, every animal was allocated randomly to one of the treatment or control groups (n = 20 for each treatment group, n = 30 controls), and pre-operative BBB (Basso, Beattie and Bresnahan locomotor score, further described below) baseline data were obtained. We defined exclusion criteria for this study based on the BBB-Score to identify animals that did not have a sufficient (BBB on day 3 > 7 or at week 1 > 13) or too strong (BBB < 10 at week 2) contusion to the spinal cord. This led to the exclusion of n = 6 control animals, n = 8 from the subacute group and n = 4 from the chronic treatment group.

### 2.2. Surgical Procedure

In this study, a spinal cord contusion injury model using the Infinite Horizon (IH) impactor (Precision Systems and Instrumentation, LLC, Lexington, KY, USA) was utilized. The rats were first exposed to a 3% isoflurane gas/air mix, which induces a short general anesthesia. They were then injected intraperitoneally with Ketasol (100 mg/kg bodyweight Ketaminhydrochloride 100 mL/mg, Dr. E. Graeub AG, Bern, Switzerland) and Rompun (5 mg/kg bodyweight Xylazine-Hydrochloride, Gamma-Hydroxybenzoacidmethylesterate 2%, Bayer, Leverkusen, Germany), according to their bodyweight, for maintaining anesthesia. Anesthetic depth was monitored through the toe pinch test, oxygen saturation, heart rate and breathing rate. If needed, additional low-dose isoflurane gas/air mix (0.5–1%) was used for deepening anesthesia. By using a rectal probe, core body temperature was measured and maintained at 37 °C through the use of an adjustable heating pad during the surgery.

Temperature support was continued until recovery from anesthesia. After wound incision, the lamina arcus vertebrae of the 11th thoracic vertebra were prepared and a laminectomy was performed by drilling a small hole, slightly larger than the 2.5 mm tip of the IH impactor, into the lamina. That hole was then slightly enlarged and cleared of bone debris using a small bone rongeur. Afterward, the animal’s spinal column was held in place by clamping it rostrally and caudally to Th11 with the impactor’s stabilizing forceps. Using the hand wheels at the front and the side, the steel rod was positioned exactly 3–5 mm cranial to the laminectomy hole. Finally, all animals were subjected to an impact with a defined force of 150 kdyn. Wound closure was performed in anatomical layers using absorbable 4/0 sutures.

All surgical procedures were performed under sterile conditions. Post-operative care was provided to all animals equally via subcutaneous injections of 10 mL of saline, analgesics and two antibiotic treatments (4 mg/kg bodyweight Carprofen/Rimadyl, Pfizer, NY, USA; 7.5 mg/kg bodyweight Enrofloxacin/Baytril Bayer, Leverkusen, Germany). This treatment was given for the first 3 days after surgery, and the rat’s bladder was manually expressed until spontaneous urination returned.

### 2.3. ESWT Setup

**Figure 1:**
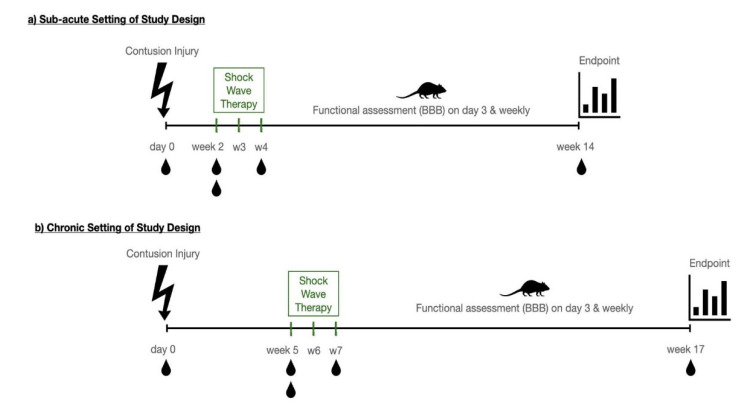
Study Design. In this study, two different timepoints were chosen to apply the shockwave therapy. Initially, a contusion injury was inducted upon laminectomy. In the subacute setting (**a**), therapeutic shockwaves were applied for three consecutive weeks, starting at week 2 after injury. In the chronic setup (**b**), the same treatment paradigm was applied starting at week 5. In both setups, the observation period until the end point was 10 weeks following the last therapeutic session. Throughout this period, functional assessment of motor function and regeneration using the BBB-Score (Basso, Beattie and Bresnahan locomotor score) was determined on day 3 and subsequently every week. Serum sampling for miRNA analysis, indicated by the schematic blood drop, was conducted prior to injury, prior to the first shock wave application, 1 h after the first wave application, immediately after the last shock wave application and at the end point.

Based on available literature [16,34,35], as well as our own preliminary data, animals randomly assigned to a therapy group received 500 impulses of focused low-energy shockwaves using an electro-hydraulic shockwave device (OrthoGold 100, MTS Medical UG, Konstanz, Germany) with an energy flux density of 0.1 mJ/mm^2^ and a frequency of 5 Hz (Focused shockwave applicator: OE50) onto the area of surgery at the 11th thoracic vertebra. Figure 1 depicts the therapeutic settings and indicated timepoints at which shockwaves were applied. In the subacute setting, therapeutic intervention was conducted in week 2, week 3 and week 4 after injury, while in the chronic setting, shockwaves were applied in week 5, week 6 and week 7 after contusion. Control group animals did not receive shockwave intervention, which was the only distinction from the treatment group animals. The observation time was chosen to include 10 weeks after the last treatment session. For each treatment, animals were introduced to a short general anesthesia using 3% isoflurane gas/air mix. The area of the laminectomy was then identified transcutaneously, and ultrasound gel was applied to the area. The shockwave applicator was then placed directly above the laminectomy site for treatment of the injured spinal cord.

### 2.4. Functional Testing—The BBB-Score

**Figure 2:**
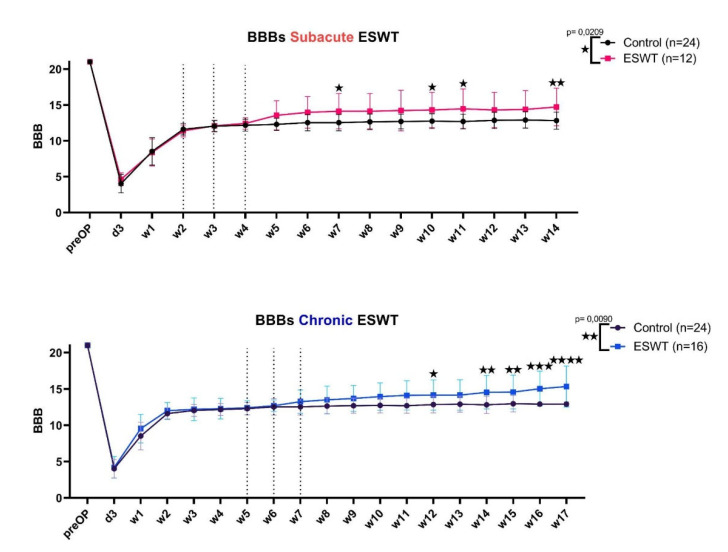
BBB-Scores (Basso, Beattie and Bresnahan locomotor score) of subacute (**a**) and chronic (**b**) ESWT (extracorporeal shockwave therapy) settings. In (**a**), the mean BBB-Scores of the subacute ESWT group (red) are compared to the control group (black). In (**b**), the mean BBB-Scores of the chronic ESWT group (blue) are compared to the control group. On the x-axis, time is plotted against the y-axis with the respective BBB-Score. Error bars indicate standard deviation. RM 2-way ANOVA was performed, and significant differences between groups are indicated by an asterisk (values with *p* < 0.05 are summarized as a single asterisk “★”, *p* < 0.01 as ”★★”, *p* < 0.001 as ” ★★★”, *p* < 0.0001 as ”★★★★”). The *p*-value for the treatment effect is shown in the top right for each experimental setting.

For evaluation of hind limb motor function recovery and coordination between fore- and hindlimbs after SCI and ESWT, we used an open field walking test, the BBB-Score, which is a well-established locomotor rating scale used in the field of spinal cord research. In short: rats were placed on an open field (in our study, we used a circular metal enclosure with a diameter of 100 cm) and each hindlimb was assessed separately using a scale from 0 to 21 while the rat moved freely for at least 4 min. The test was conducted by two independent blinded observers before injury on day 3 post-injury and once per week throughout the observation period. The mean values of left and right hind limb scores were used to analyze changes before and after treatment (Figure 2). After applying predefined exclusion criteria, a total of n = 24 control animals, n = 16 chronic treatment group and n = 12 for the subacute treatment group was achieved.

### 2.5. Histology and Morphometric Analysis

#### 2.5.1. µ-CT

In order to evaluate the three-dimensional appearance of spinal cords after the respective observation periods, spinal cords bearing contusion injury were extracted with the bone of the spinal column intact. The spinal column was stained in 50 mL centrifuge tubes filled with 1% Lugol’s Iodine for 48 h at 4 °C on a roller mixer. If the staining duration was determined to be insufficient (as evaluated via overview scans), the staining was continued in 24 h increments, replacing the staining solution each time, until the spinal cord was fully stained. Scans were performed in a SCANCO µCT 50 (SCANCO Medical AG, Brüttisellen, Switzerland) in the residual staining solution at 90 kVp with 200 µA with a 0.5 mm Al Filter. The 1000 Projections/180° were integrated for 500 ms with a FOV of 35.2 mm. The scans were reconstructed to an isotropic resolution of 17.2 µm. A height of approximately 27 mm was scanned with the defect in the center of the scan (Supplementary Figures S1 and S2). Subsequently, scans were converted to dicom slices and evaluated using the Fiji software (ImageJ v1.51h) and Definiens Developer XD 2.7 (Definiens AG, Munich, Germany). In Fiji, stacks were cropped to the relevant region of the spine and a mask was drawn marking the outer surface of the spinal cord using the polygonal selection tool and the interpolation tool in the ROI manager. Finally, a specifically developed Definiens ruleset was created to automate measurements of various parameters at the injury site, including cavity size and spared white matter, as described in detail in Appendix A.

#### 2.5.2. Immunohistochemistry

After µCT scanning, histological and immunohistochemical analyses were performed. Lugol’s iodine solution was washed out using 70% ethanol. Samples were rinsed in water for one hour and then decalcified in 10% EDTA for several weeks. The correct position for the histological sections was obtained with the help of µCT images. Embedding in paraffin was performed via the intermedium xylene after dehydration of the samples in an ascending ethanol series, beginning with 50% ethanol. Samples were cut into 4 µm thin sections and dried in a 37 °C incubator overnight. Standard H&E staining was performed as well as staining for Neurofilament (NF) and Luxol Fast Blue stainings.

Luxol Fast Blue staining was performed with a Luxol staining solution in 96% EtOH (Waldeck). After that, the sections were differentiated with lithium carbonate, which results in a crisp myelin staining in blue. Then, the Nissl bodies were stained with cresyl violet (Carl Roth, Karlsruhe, Germany).

For NF staining, slides were steamed for 20 min in sodium citrate buffer (pH = 6) for 20 min. Afterward, sections were incubated with Bloxall (VectorLabs) for 10 min. The incubation with primary antibodies (Neurofilament, Clone2F11, Dako, Santa Clara, CA, USA) was conducted in a humified chamber at room temperature for 1 h. Secondary antibodies, anti-mouse (BrightVision poly HRP), were incubated for 30 min, and the detection of the signal was obtained using ImmPACT Nova Red (VectorLabs). Finally, all sections were counterstained using Mayer’s Hematoxylin and permanently embedded.

For analysis, slides were scanned with the Vectra Polaris Automated Quantitative Pathology Imaging System (Akoya Biosciences, Marlborough, MA, USA) and evaluated with HALO v3.4.2986.170 software (Indica Labs, Albuquerque, NM, USA). The Multiplex IHC v3.0 module was used for NF staining assessment. Representative areas were marked to generate a random forest classifier for analysis of Luxol stained slides.

### 2.6. MicroRNA Sampling and Analysis

In order to evaluate changes in the miRNA expression profile in blood serum of shockwave-treated animals, we screened for differential expression of 187 miRNAs in 8 animals (n = 4 for the subacute, and n = 4 for the chronic treatment group). We used samples of the same animal before and 1 h after ESWT in both treatment groups. These miRNAs were determined by researching keywords such as inflammation/anti-inflammatory, angiogenesis, neuronal development/expression/enrichment/protection, motor function, and overall good detection in serum. Promising candidates for further validation in our experimental setup were chosen according to the *p*-value and effect size (log2 fold change). The new miRNA-arrays consisted of 21 promising candidates (each treatment group had a slightly different array with 11 common miRNAs), which were analyzed at 5 different timepoints: preinjury, pre-treatment (week 2 for the subacute group and week 5 for the chronic), 1 h after the first treatment, 1 h after the third treatment before sacrificing the animals. Blood sampling for the controls was performed at the corresponding timepoints.

#### 2.6.1. Serum RNA Extraction

Total RNA was extracted from 100 µL serum using the miRNeasy Mini Kit (Qiagen, Hilden, Germany). Samples were thawed on ice and centrifuged at 12,000× g for 5 min to remove any cellular debris. For each sample, 200 µL of serum was mixed with 1000 µL Qiazol and 1 µL of a mix of 3 synthetic spike-in controls (Qiagen, Hilden, Germany). After a 10-min incubation at room temperature, 200 µL of chloroform was added to the lysates, followed by cooled centrifugation at 12,000× g for 15 min at 4 °C. Precisely 650 µL of the upper aqueous phase was mixed with 7 µL glycogen (50 mg/mL) to enhance precipitation. Samples were transferred to a miRNeasy mini-column where RNA was precipitated with 750 µL ethanol followed by automated washing with RPE and RWT buffer in a QiaCube liquid handling robot. Finally, total RNA was eluted in 30 µL of nuclease-free water and stored at −80 °C to await further analysis.

#### 2.6.2. MicroRNA Reverse-Transcription Quantitative PCR Analysis in Serum RNA (RT-qPCR)

Starting from total RNA samples, cDNA was synthesized using the miRCURY LNA RT kit (Qiagen, Hilden, Germany). Reaction conditions were set in accordance with the manufacturer’s specifications. In total, 2 µL of total RNA was used per 10 µL reverse transcription (RT) reaction. To monitor RT efficiency and presence of impurities with inhibitory activity, a synthetic RNA spike-in (cel-miR-39-3p) was added to the RT reaction. PCR amplification was performed in a 384-well plate format in a Roche LC480 II instrument (Roche, Germany) using a miRCURY LNA SYBR Green PCR kit (Qiagen, Hilden, Germany) with the following settings: 95 °C for 10 min, 45 cycles of 95 °C for 10 s and 60 °C for 60 s, followed by melting curve analysis. To calculate the cycle of quantification values (Cq-values), the second derivative method was used. Spike-in control values were used for monitoring data quality. Serum samples with low RNA recovery assessed by spike-in levels were not included in the analysis. Data normalization was performed using the RNA spike-in controls by subtracting the individual miRNA Cq-value from the RNA Spike-In Cq-value, thus obtaining delta-Cq (dCq) values that were used for the analysis. Hemolysis was assessed in all samples using the ratio of miR-23a-3p versus miR-451a, and a cut-off of dCq > 7 was applied to indicate a high risk of hemolysis [36].

Missing values were imputed with 42, representing the lowest detectable amount of miRNA in the sample, before normalization. After imputation, outliers were identified via Grubb’s test at a significance level of 5 % and excluded. Furthermore, serum samples with a Uni-Spike-In 4 Cq value more than 3 standard deviations above the arithmetic mean of all the other samples were excluded due to low RNA recovery. This led to the exclusion of the pre-surgery sample of individual 91 (control group), which was then imputed with the arithmetic mean of the Cq values of the other individuals in the same experimental group, setting, timepoint and miRNA because otherwise only two values would remain in that experimental group, and because the pre-surgery Cq-values can be reasonably assumed to be similar.

#### 2.6.3. Principal Component Analysis (PCA) of miRNAs

In order to identify the miRNAs with the greatest fluctuations and therefore to gather potential insights into the most important ones in regard to SCI and ESWT, we performed PCA on the miRNA RT qPCR across groups and timepoints. After removing all rows (sample-timepoint combinations) with missing values, a PCA of baseline normalized miRNA ∆Cq values was performed via the prcomp() function from the stats package in R [37] with centering, because PCA requires it, but without scaling, as the data are all on the same scale and potentially valuable information would be lost [38].

Based on the first few principal components cumulatively describing at least 80% of total variance, the relative feature importance of each miRNA was calculated as follows:

#### 2.6.4. Hypothesis Tests for Diagnostic miRNAs and Treatment Effect

In the interest of finding miRNAs most closely related to SCI, we performed hypothesis tests on the difference between miRNA levels—as quantified via RT qPCR—at the pre- and post-surgery timepoints. These diagnostic hypothesis tests were performed using the wilcox.test() function from the stats package in R [37]. These tests were paired non-exact Wilcoxon signed-rank tests, in which two samples from the same animal but different timepoints were compared. Non-exact tests were chosen out of necessity, as the exact tests failed due to ties. Based on these diagnostic tests, volcano plots highlight the miRNAs most significantly changed by the injury.

A repeated measures two-way ANOVA was performed to assess the treatment effect and differences between ESWT and control groups at individual observational timepoints. Geisser Greenhouse and Šidák corrections were applied to correct sphericity and multiple comparisons. In the case of missing values, a mixed-effects model was automatically applied using Graphpad.

#### 2.6.5. Correlation of Functional Scoring and miRNA Levels

Spearman’s correlation coefficient between BBB-Scores and miRNA serum levels at the respective weeks was calculated with Graphpad Prism 9.2.0. Analysis was performed separately for the ESWT treated and control group, as well as for the data from both groups combined. Additionally, for miRNAs analyzed in subacute and chronic treatment settings, pooled data were used for assessment.

## 3. Results

### 3.1. ESWT Significantly Improved Motor Function after SCI

In this study, we evaluated the effects of ESWT on functional regeneration in subacute and chronic SCI. Throughout the observation period after injury and treatment, continuous assessments of changes in motor function were conducted to observe the effects of ESWT on functional regeneration. We found that immediately after treatment with ESWT, rats of both treatment groups experienced an improvement in coordination, better paw positioning and toe clearance, whereas control animals, except for one outlier, reached a plateau of BBB = 12–13 and did not improve further (Figure 1). Animals treated at a subacute timepoint after SCI reached a mean BBB-Score of 14.7 points ± 2.6 at the end of observation time, and those treated at a chronic stage reached a mean score of 15.6 points ± 2.6, whereas control animals had a mean score of 12.9 points ± 0.3. This difference between groups was greater in the chronic treatment setup, where rats seemed to improve until the end of observation time with statistical significance compared to control, starting at week 10 until week 17 after SCI. For the subacute treatment, statistical significance was reached at week 7, 10, 11 and 14 after contusion injury.

### 3.2. Calculated 3D Parameters Generated by µCT-Scans Did Not Show Significant Morphological Differences between Therapy and Control Groups

At the end of the observation period, we aimed to analyze structural and dimensional parameters. In order to account for the spatial complexity of secondary spinal cord injuries, we implemented three-dimensional imaging of the contused spinal cord using ex-vivo µCT. μCT is widely used for the study of calcified tissue, but a similar use for soft tissues is hindered by their low X-ray attenuation. This limitation was overcome in this study by the use of Lugol’s solution as contrast agents [39]. Outcome parameters, such as cystic volume and remaining healthy tissue cross sections, were used to quantify 3D morphological changes in spinal cords of the control and therapy groups.

**Figure 3:**
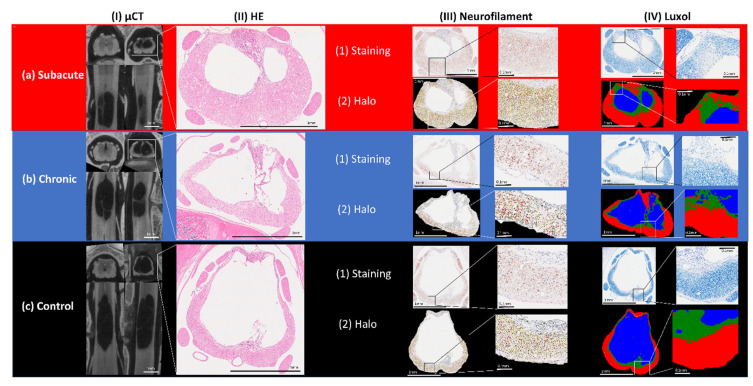
Morphological evaluation of injured spinal cords. (**a**) from left to right: two longitudinal sections of respective µCT scans (cranial (**left**) and sagittal (**right**) plane) as well as cross-sections of spinal cords rostral of the injury (**left**) as well as at the site of contusion (**right**). Representative spinal cords of groups (**a**) subacute ESWT (extracorporeal shockwave therapy), (**b**) chronic ESWT and (**c**) control: Corresponding subsequent HE staining (hematoxylin/eosin) of the injured area; (**a**–**c**) (1) NF-staining (neurofilament) and Luxol staining of the same spinal cord slice and (**a**–**c**) (2) automated identification of NF-positive and Luxol-positive areas using Halo software. Far right: red area indicates myelinated tissue, green area indicates demyelinated tissue and blue indicates the cystic cavity.

**Figure 4:**
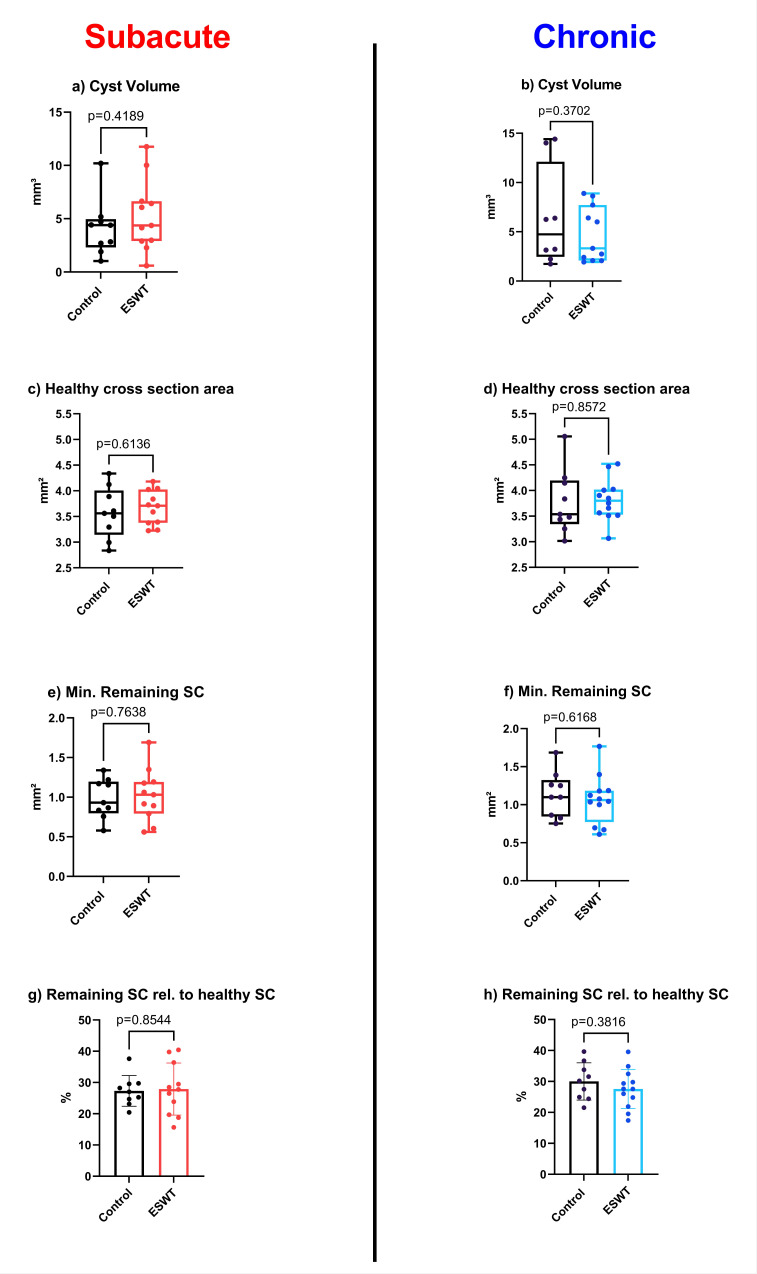
Calculated parameters generated by µCT-Scans. Various parameters were calculated using an algorithm to explore the potential morphological effects of therapeutic intervention in a subacute ((**a**,**c**,**e**,**g**), ESWT group in red and controls in black) as well as chronic ((**b**,**d**,**f**,**h**), ESWT group in blue and controls in black) setting. The cystic volume (**a**,**b**) was determined in clear distinction from a syrinx. Healthy cross-sectional area (**c**,**d**) was determined distal to the spinal cord defect site, while the minimal remaining spinal cord (**e**,**f**) was measured as a plane at the maximal diameter of the cystic cavity. Remaining spinal cord relative to healthy spinal cord (**g**,**h**) was calculated in comparison to the healthy cross section. **Cystic volume** = total volume of cyst from 5 mm caudal to 5 mm rostral of the defect. **Healthy cross section area** = average cross-sectional area at a distance of 5–10 mm rostral and caudal of the lesion area of a linear regression of healthy tissue outside the defect area to estimate the healthy cross-sectional area. **Minimal remaining spinal cord** = smallest remaining cross-sectional area of healthy spinal cord tissue. **Remaining spinal cord relative to healthy spinal cord** = smallest remaining cross-sectional area of healthy spinal cord tissue as a percentage of the healthy reference.

In Figure 3, representative samples of µCT scans show longitudinal slices as well as cross-sections of spinal cord tissue after injury compared to histological evaluation. Both control groups included n = 9 samples each, while the subacute treatment setting included n = 11 and the chronic treatment group included n = 12 samples. Delineation of the cystic cavity and syrinx was determined by automated calculation. Extracted parameters were then compared in regard to regenerative potential. Volumetric differences between ESWT-treated and control groups were not significant, as shown in Figure 4.

### 3.3. Histological and Immunohistochemical Analysis Did Not Reveal Significant Differences after ESWT in Comparison with Control Group

**Figure 5:**
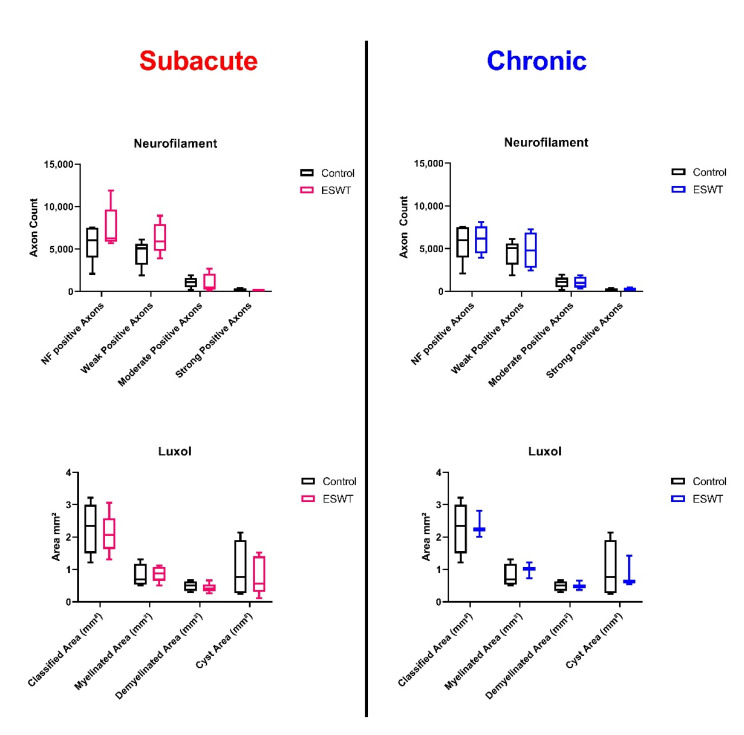
Box and whisker plots of histological stainings. In Figure 5, box and whisker plots (minimum to maximum, with Q1, Median and Q3) illustrate the differences between control (black) and chronic (blue), as well as subacute (red) treatment groups.

In order to complement 3D µCT-derived morphological findings, histology and immunohistochemistry of spinal cord tissue were conducted and compared. Analysis revealed significant loss of axons as well as demyelination in the affected area of the spinal cord following contusion. A general reduction in spinal cord diameter was noticeable, as already observed in the μCT evaluation. Trained algorithms for the automatic detection of NF-positive axons as well as Luxol-positive myelin performed with a high accuracy and enabled quantitative analysis of the histological slides (Figure 3). Comparison of treatment vs. control groups, however, did not reveal significant differences, neither in standard HE staining nor in neurofilament and Luxol stainings (Figure 5).

Contrary to observed functional improvement and previously published data, we did not observe any significant morphological differences, e.g., spared white matter, after ESWT in either treatment group compared to controls. This finding was consistent with the aforementioned automatically measured spinal cord parameters in µCT imaging data, indicating an underlying effector mechanism of ESWT in addition to neuroprotective or regenerative mechanisms.

### 3.4. ESWT Significantly Influenced the Expression Patterns of Systemic miRNA

**Figure 6:**
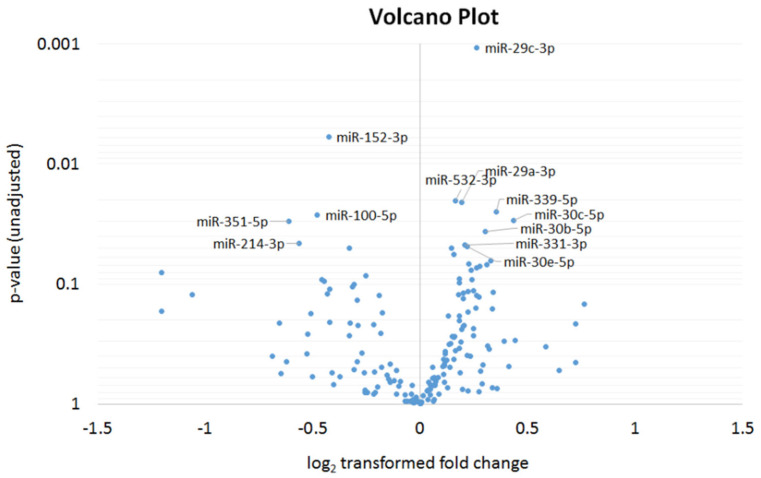
MicroRNA screening. Results of a pairwise comparison of circulating microRNA levels before and after shockwave treatment. Volcano plot shows the relationship between the log2 fold change in 187 microRNAs between treated and untreated animals. All miRNAs with *p* < 0.05 are labeled. Statistical differences were calculated using a paired two-sided *t*-test.

In order to elucidate underlying mechanisms after SCI and ESWT, we performed an analysis of systemic microRNAs and their potential contributions to regeneration at defined timepoints of the observation period. The initial screening for ESWT-associated changes in miRNA profiles (Figure 6) included 187 miRNAs and resulted in a panel of 21 miRNAs. This panel was based on a *p*-value (<0.05) and effect size (log2 fold change > ±0.5 and *p*-value < 0.1) and effect size (log2 fold change) before and after ESWT for both treatment setups for further validation. Out of these, 11 miRNAs were common to both arrays.

#### 3.4.1. Several Identified miRNAs Showed Potential Therapeutic Relevance in Either Subacute or Chronic Experimental Setting

In the subacute experimental setting, seven potentially therapeutically relevant miRNAs were identified. Differences between the ESWT treatment and control group for pooled timepoints (column factor in two-way ANOVA) were observed for hsa-miR-100-5p and hsa-miR-136-5p. Furthermore, rno-miR-148b-5p, rno-miR-214-3p, hsa-miR-28-5p, hsa-miR-218-5p, hsa-miR-199a-3p and hsa-miR-375 showed differences between groups at repeated measures corrected weekly compared to after correction for repeated measures. Diagnostic markers, in terms of statistically significant differences between baseline values and miRNA expression levels at week two, were observed for hsa-miR-199a-3p, hsa-miR-375 and hsa-miR-152-3p.

In the chronic experimental setting, four miRNAs were potentially therapeutically relevant. These include hsa-miR-100-5p, hsa-miR-124-3p, hsa-miR-218-5p, with a significant difference between groups at the endpoint and hsa-miR-124-3p as well as hsa-miR-382-5p with significant differences between pooled timepoints. Additionally, nine miRNAs were identified as possible diagnostic markers, including hsa-miR-218-5p, hsa-miR-125-5p, hsa-miR-133b, hsa-miR-144-3p, hsa-miR-152-3p, hsa-miR-199a-3p, hsa-miR-29c-3p, rno-miR-148-5p and rno-miR-381-3p.

**Figure 7:**
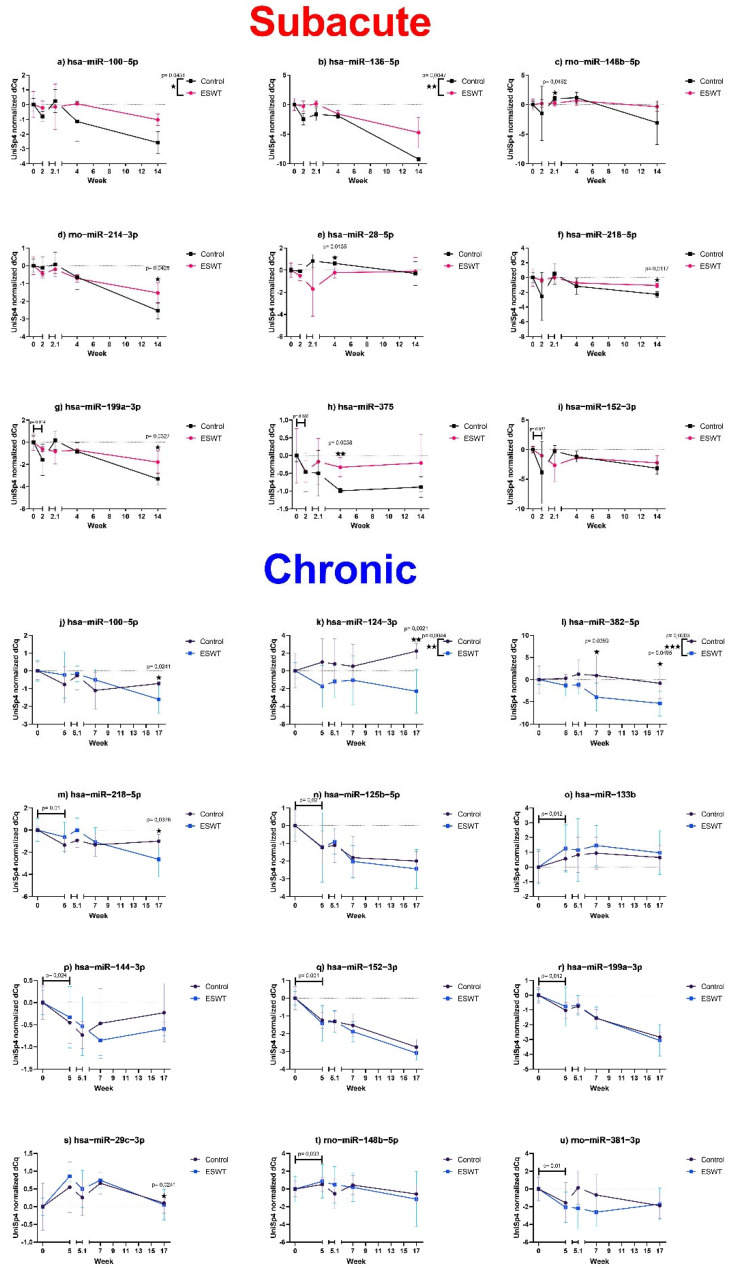
Longitudinal miRNA expression profile. Figure 7 shows the mean and standard deviation of baseline normalized miRNA serum levels of control (black) and chronic (**a**: blue), as well as subacute (**b**: red) treatment groups plotted against time. Note that the x-axis is stretched between the timepoints of the first treatment and 1 h after the first treatment (labeled as timepoints 2.1 and 5.1 for subacute and chronic experimental settings, respectively) for improved visualization of acute treatment effects. Significant differences in weekly mean (RM 2-way ANOVA) are indicated by an asterisk, and significant overall treatment effects are indicated by an asterisk and the *p*-value at the top right of the corresponding graph (values with *p* < 0.05 are summarized as a single asterisk “★”, *p* < 0.01 as ”★★”, *p* < 0.001 as ” ★★★”). Diagnostic markers (pre-OP versus first observational timepoint) are marked by a line with the respective *p*-value.

Corresponding *p*-values as well as the mean and standard deviation over time are reported in Figure 7.

#### 3.4.2. A Sub-Set of miRNAs Correlated with Functional Outcome

In order to investigate the influence of the examined microRNAs on motor function, Spearman’s correlation coefficients were calculated. Correlation coefficients r > 0.5 were observed in the pooled data from subacute and chronic control groups (ESWT and control combined) for hsa-miR-29c-3p (r = 0.63, *p* = 0.0003) in the subacute ESWT group for hsa-miR-375 (r = 0.50, *p* = 0.0125) and in the control group for hsa-miR-146a-5p (r = 0.56, *p* = 0.0306) and hsa-miR-29c-3p (r = 0.52, *p* = 0.0466). In the chronic treatment set up, no correlations with r > 0.5 were observed.

#### 3.4.3. PCA of microRNA Expression Data Revealed Highest Relative Feature Importance of miRNA 382-5p across Both Treatment Models

**Figure 8:**
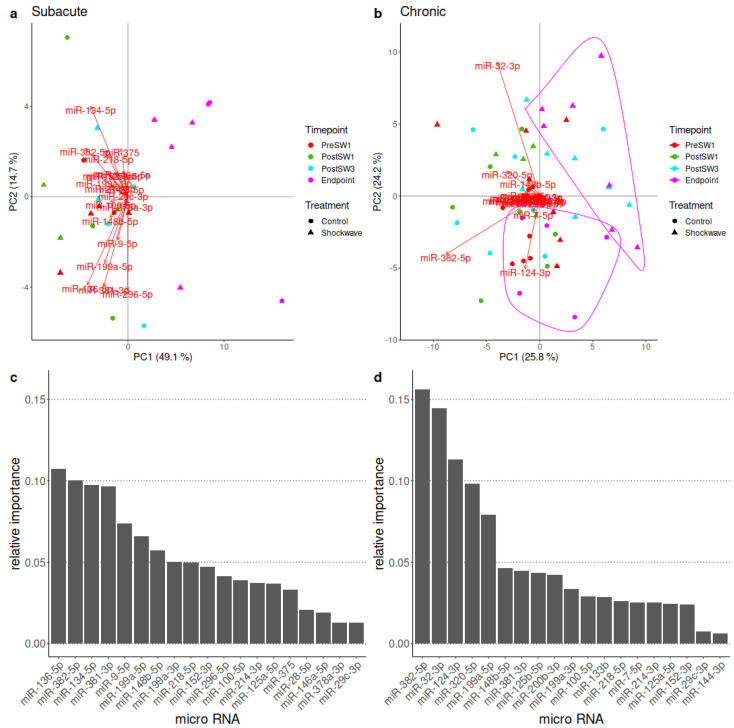
PCA (principal component analysis) results of miRNA RT qPCR. (**a**,**b**) PCA biplots. Vectors are loadings of the miRNAs, while the points are the samples in the coordinate system made up of the first two principal components. (**a**) Subacute biplot, (**b**) chronic biplot; apparent clusters of endpoint samples encircled, (**c**) relative feature importance of the subacute setting, (**d**) relative feature importance of the subacute setting.

PCA results of the miRNA RT-qPCR data are shown in Figure 8. In the subacute setting, no miRNAs stood out, and there were no obvious clusters of samples. The most important miRNAs, based on the first four PCs, which describe over 80% of total variance, are miR-136-5p, miR-382-5p, miR-134-5p, mir-381-3p and miR-9-5p.

In the chronic biplot, the endpoint samples form two clusters distinguished by the therapy group. The most fluctuating miRNAs according to the biplot are miR-32-3p, miR-382-5p and miR-124-3p, which aligns with the calculated feature importances, where those are the first three, followed by miR-320-5p and miR-199a-5p (Figure 8).

## 4. Discussion

In this study, we investigated the effects of subacute and chronic ESWT after SCI. For that purpose, we used a contusion spinal cord injury model in the rat and introduced low-energy extracorporeal shockwave treatment either at week 2 or week 5 after SCI for three consecutive weeks. Functional outcome, morphological changes, and the effect of ESWT on systemic microRNA expression profiles were assessed.

To our knowledge, there is still very limited data for the effects of ESWT in subacute and chronic timepoints after SCI. Our study aimed to provide further evidence on the efficacy of ESWT after SCI, building on previous promising observations of ESWT in acute trials [16,35,40].

Here, we report a significant beneficial effect of this non-invasive therapy on the functional outcome of subacutely and chronically treated animals after spinal cord contusion injury. This improvement in motor function was not reflected by morphological changes between controls and ESWT-treated groups according to the 3D µCT histological parameters. However, miRNA expression analysis provides new insights into the underlying mechanisms of the functional improvement observed after ESWT. Several studies have described the release, modulation and therapeutic implications of RNAs after ESWT in various pathologies and tissues [16,33,35,41,42]. A study conducted by Yamaya S et al. showed that shockwave treatment in a model of acute spinal cord injury three times a week for three consecutive weeks was able to significantly increase mRNA and protein expression of VEGF in a rodent contusion model. Furthermore, neuronal cell loss was significantly reduced along with improved locomotor function [16].

A previous publication described aortic cross clamping in mice causing severe ischemic injury to the spinal cord, thus leading to consecutive paraplegia. Shockwave therapy was applied immediately after injury resulted in decreased neuronal degeneration as well as improved motor function and survival [11].

Another study was conducted by our research team on the effects of ESWT in a sciatic 8 mm autologous nerve transplantation model in the rat. Shockwave-treated animals showed improved regeneration and ameliorated functional performance of the treated limbs compared to untreated controls [7]. Furthermore, a study by Schuh et al. indicated that ESWT has a strong effect on the repair phenotype of Schwann cells in vitro [14].

We were also able to contribute to the understanding of the underlying mechanisms associated with shockwave therapy in a previous study. We demonstrated that energy and pulse-number-dependent ATP release triggers the expression of ERK 1/2 and MAPK, which results in enhanced cell proliferation in different cell lines. Purinergic signaling-induced Erk1/2 activation was found to be essential for this proliferative effect. This was further confirmed by an in vivo study in a rat wound healing model, in which shockwave-treatment-induced proliferation and increased wound healing in an Erk1/2-dependent fashion were observed [12].

Further beneficial effects of ESWT after SCI could potentially be due to changes in the level of expression of miRNAs. Indeed, in this current study, we identified several significant changes in circulating miRNAs, associated with neurode-/re-generation (Figure 7).

The ESWT-treated group presented increased serum levels of miR-100-5p in the subacute setting, in contrast to the chronic group. Therefore, our findings regarding miR-100-5p in response to ESWT remain inconclusive.

Long-term effects of miR-136-5p, which showed a statistically significant increase in the ESWT group, could be beneficial for regaining motor abilities, through SH3GL Interacting Endocytic Adaptor 1 (SGIP1) [43] altered synaptic plasticity [44,45]. Furthermore, in vitro experiments by Peng et al. suggested an association between the increase in the SCI-related miR-136-5p and A2O protein (TNFα-induced protein 3) reduction and following inflammatory response after IL-1β, IL-6, TNF-α, IFN-α, IKKβ, and NF-κB upregulation. This response could worsen injury outcome [46], as confirmed in two in vivo trials, which assessed BBB-Scores one day and up to 7 days after SCI [47,48]. Furthermore, upregulation of the stem loop sequence miR-136 showed TIMP Metallopeptidase Inhibitor 3 (TIMP3)-associated reduction in cell apoptosis in ischemic SCI [49]. ESWT-induced upregulation of miR-136-5p could be part of the explanation for the beneficial functional effects of ESWT.

Another identified miRNA is miR-214-3p. Controversy exists in the literature about the role of miR-214-3p in neuroprotection. Yuanzhi Fan et al. showed neuroprotective effects after the application of tetramethylpyrazine in connection with the downregulation of miR-214-3p in SCI after 21 days [50]. In accordance with these findings, we saw a gradual decrease in miR-214-3p levels over time. However, at week 14, the ESWT-treated group showed significantly higher serum levels of miR-214-3p, as compared to control, which is supported by neuroprotective effects after upregulation of miR-214-3p, previously shown in multiple models [51,52]. Furthermore, elevated miR-214-3p levels have been associated with Quaking-related increased plasticity [53], progenitor cell differentiation in the central nervous system [54] and CSF-1-related reduced neuropathic pain [55].

In the subacute treatment setting, miR-218-5p was significantly upregulated at the end point, which is in agreement with decreased dopaminergic neuron loss in Parkinson’s disease patients due to miR-218-5p targeting LASP1 [56]. In contrast, downregulation of miR-218-5p has been associated with increased inflammatory response in patients with multiple sclerosis [57].

Significant downregulation of miR-199a-3p after SCI constitutes this miRNA as a potential marker for SCI, as also observed by other authors [58,59,60]. Furthermore, miR-199a-3p was upregulated at week 14, which indicates the beneficial effects of ESWT. It has been described previously that miR-199a-3p and 145-5p delivered through hUC-MSCs increased motor function through anti-inflammatory effects and neurite outgrowth-enhancing p-Akt/p-Erk pathway activation and NGF [61].

In response to SCI, miR-375 was downregulated at week two, making it a potential candidate for a diagnostic marker after SCI. Furthermore, directly after the first ESWT application, as well as at all successive timepoints, miR-375 was upregulated in the ESWT group. Various pathways have been described for motor neuron development in the spinal cord in response to miR-375 expression, such as targeting the CCND2 and PAX6. Additionally, neuroprotective effects of miR-375 through p53 inhibition have been described in patients suffering from spinal muscle atrophy [31]. In another study, miR-375 was shown to target Sp1 and Tle4, resulting in enhanced GnRH secretion [62], well known for its beneficial role after SCI [63,64,65,66], even in chronic SCI patients [67].

Another potential diagnostic marker, miR-152-3p, which was significantly downregulated after SCI in our study, has already been documented by Ding et al. [68].

In the chronic experimental setting, we identified eight miRNAs (miR-218-5p, miR-125b-5p, miR-144-3p, miR-152-3p, miR-199a-3p, miR-29c-3p, miR-148b-5p, miR-381-3p), which were significantly downregulated after SCI, and one (miR-133b) which was upregulated. After treatment with shockwaves, four miRNAs (miR-100-5p, miR-124-3p, miR-382-5p and miR-218-5p) were significantly altered in their expression profile.

According to the literature, miR-100-5p has previously been described to be involved in the pathogenesis of neurodegenerative diseases [69,70,71,72,73]. Wallach et al. were able to identify miR-100-5p as an endogenous toll-like receptor 7/8 ligand in cortical neurons, leading to apoptosis and neurodegeneration [72]. In the chronic setting of our study, we saw continuously decreasing miR-100-5p levels after treatment at week 5, which resulted in significantly lower expression levels at the end of observation time compared to control.

Levels of circulating miR-124-3p were significantly lower in the ESWT group compared to the control group. miR124-3p has widely been researched for its involvement in several pathologies of the CNS [74,75,76,77,78,79,80,81,82,83,84,85,86,87]. Studies have found that increased expression of miR-124-3p in microglial exosomes alleviated neurodegeneration, inhibited neuronal inflammation and contributed to neurite outgrowth, reduced inflammation and eventually promoted functional recovery in different animal models [74,79,80,82,83,84,85,86]. Vuokila et al. observed that higher levels of miR-124-3p at an acute timepoint correlated with a larger lesion area after traumatic brain injury, whereas Rainer et al. described miR-124-3p as a prognostic marker with higher expression levels leading to a worse clinical outcome after acute stroke and larger lesion areas on CT scans [75,76,83]. In contrast, studies in traumatic brain injury (TBI) models showed that miR-124-3p was persistently downregulated even up to 3 months after TBI. This has been described to be a common phenomenon after brain injury [75,76]. Most studies describe a beneficial effect of overexpressed miR-124-3p. Li et al. found that highly expressed exosomal miR-124-3p reduced cell apoptosis and ameliorated ischemia/reperfusion-induced tissue damage and nerve injury in the spinal cord [78].

One of the most promising miRNAs in our study was the miR-382-5p. While the control animals demonstrated unaltered expression levels throughout the entire experiment, animals in the ESWT group experienced a significant downregulation by the time of the third shockwave treatment, with this effect being even more pronounced at the end of observation time. In a study by Xiang et al., an abnormal downregulation of lncRNA Ftx and Nrg1 and upregulation of miR-382-5p after SCI was observed, which contributed to the inflammatory response in microglial cells and affected SCI repair [32]. Phosphatase and tensin homolog (PTEN) is a protein phosphatase and functions as a tumor suppressor in negatively regulating Akt signaling, thereby reducing cell migration, proliferation and cell growth. In a model of age-related macular degeneration, miR-382-5p has been described to negatively regulate PTEN and subsequently activate the AKT/mTOR pathway, whereas NR3C1 acted as a miR-382-5p sponge, consequently regulating this pathway [88]. Reduced PTEN and subsequent dysregulation of the AKT/mTOR pathway have also been linked with neurodegenerative diseases, such as Alzheimer’s as well as syndromic autism spectrum disorder. In another study of chronic unpredictable mild stress in rats, upregulation of miR-382-5p and downregulation of NR3C1 were observed in rats’ hippocampi. miR-382-5p targeted NR3C1 and inhibited the expression of NR3C1 in rats’ hippocampi, leading to more severe and higher prevalence of depression with an elevated latency to feed and immobility time. Inhibition of miR-382-5p had the opposite effect [88]. Li et al. found that NR3C1 downstream targets BDNF and p-TrkB were also oppositely regulated to miR-382-5p in rats’ hippocampi [89]. BDNF has been widely described as being associated with increased plasticity and, therefore, functional recovery after SCI [90]. The observed reduction in miRNA-382-5p levels after ESWT in our study indicates a potential beneficial effect via this axis.

In our chronic experimental setting, miR-218-5p was found to be significantly downregulated at the end of the observation time [91]. MmiR-218-5p expression was significantly elevated in the blood of patients with acute ischemic stroke [92], whereas, in another study, downregulation of miR-218-5p protected against oxygen-glucose deprivation/reperfusion-induced injury of PC12 cells. There, a neuronal cell model for acute ischemic stroke was used to reduce inflammatory cytokine secretion, oxidative stress, cell apoptosis and maintenance of endovascular homeostasis via upregulating NDRG4. Several diagnostic miRNA markers for spinal cord injury have already been described in the literature [57,61,68,93,94,95,96]. In our study, we found miR-218-5p, miR-199a-3p, miR-29c-3p, miR-133b to be similarly up- or downregulated following spinal cord injury.

These findings, in conjunction with our own data, could provide insight into possible underlying mechanisms leading to observed functional recovery after SCI and ESWT treatment at subacute and chronic timepoints post-injury.

However, limitations of this study have to be noted. Our explorative study did not include a sham operated group. Furthermore, functional improvement within the chronic setting was still ongoing by the end of the observation period, which led to the suggestion of a prolonged phase of observation in future studies. Furthermore, further investigations are required to explore the effects of the laminectomy on miRNA changes, as improved bone regeneration due to ESWT could also contribute to some of the observed changes in miRNA levels. Substantial financial funding is required to advance miRNA analysis with a larger number of implications. Due to limited miRNA recovery in some samples during qPCR, some values needed to be excluded from the analysis.

## 5. Conclusions

This study demonstrates the functional improvement of ESWT-treated animals after SCI in a subacute and chronic treatment setting. Our results suggest that this functional improvement was not based on morphological changes. However, significant changes in circulatory levels of miR-382-5p and miR-375 upon ESWT were observed. This could provide further insights into underlying mechanisms regarding their influence on neuroplasticity as well as inflammatory response, hence potentially providing targets for therapeutic intervention in future studies. Results of this study were fundamental for a recently started two-arm three-stage adaptive, prospective, multi-center, randomized, blinded, placebo-controlled clinical trial on the effects of ESWT in acute traumatic spinal cord injury (ClinicalTrials.gov Identifier: NCT04474106).

## Data Availability

The authors confirm that the data supporting the findings of this study are available within the article and its Supplementary Materials. Raw data supporting the findings of this study are available from the corresponding author [D.H.] on request.

## Supplementary Materials

The following supporting information can be downloaded at: https://www.mdpi.com/article/10.3390/biomedicines10071630/s1, Figure S1: Visualization of spinal cord injury using contrast-enhanced μCT, Figure S2: Overview of µCT scans, Figure S3: Volcano Plots of diagnostic hypothesis tests of miRNA RT qPCR data

## Appendix A

Due to the variability of staining intensity, thresholds have to be chosen depending on the intensity of individual samples. To determine the thresholds, the median intensity in the tissue of the spinal cord on the first and last four slices, as well as the median intensity in the void between the spinal cord and bone, were measured. Thresholds were set relative to these measurements (Median Void + (Median Spine − Median Void) × 0.4).

The manually drawn surface of the spinal cord was corrected by several pixelwise growing steps. Limited by a threshold, the spinal cord was grown up to 5 pixels into the void and the void up to 5 pixels into the spinal cord using a temporary class. The spinal cord was then grown up to 10 pixels into this temporary class limited by the same threshold and a surface tension constraint (9 × 9 × 3 ≥ 0.45), followed by a single growing step without a threshold but a more restrictive surface tension (5 × 5 × 3 ≥ 0.5), which was repeated in the other direction. The remaining temporary class was assigned to the void. The result is a smoother segmentation of the spinal cord, which more closely follows a specific intensity value than the manually drawn mask.

Next, the spinal cord is segmented into tissue and cyst. The void is coated 3 pixels into the spinal cord with a temporary class to exclude the outer surface from segmentation. The remaining spinal cord is then thresholded in the tissue and cyst. The segmentation is smoothed using a series of pixel-wise growing steps. The tissue is grown up to 3 pixels into the cyst and back without constraint and then a single step with a surface tension (5 × 5 × 3 ≥ 0.55) and back (5 × 5 × 3 ≥ 0.45). The cyst is then grown in the Z plane with a surface tension (5 × 5 ≥ 0.4) into the temporary class. The remaining temporary class is assigned to the tissue. Finally, the cyst is grown up to 10 pixels with a surface tension (7 × 7 × 3 ≥ 0.65).

The volume of the tissue and cyst is exported in eight slice increments.
