# Supplementary material for: Effects of Extracorporeal Shockwave Therapy on Functional Recovery and Circulating miR-375 and miR-382-5p after Subacute and Chronic Spinal Cord Contusion Injury in Rats
Source: Biomedicines. 2022 Jul 7;10(7):1630. doi: 10.3390/biomedicines10071630 (PMC9313454; doi:10.3390/biomedicines10071630)
Supplement: Supplementary file 1 [file biomedicines-10-01630-s001.zip › Supplementary Figure descriptions.pdf]

Supplementary Figure S1: Visualization of spinal cord injury using contrast-enhanced µCT . Levels of cross sections are marked by green arrows and are depicted on the right side of each coronal section. Automated calculation was performed by differences in grey-values. Margins of cavity formation are indicated by yellow lines.

Supplementary Figure S2: Overview of µCT scans axial as well as longitudinal sections and cross sections of uninjured rostral and injured contused areas

Supplementary Figure S3: Volcano Plots of diagnostic hypothesis tests of miRNA RT qPCR data. Timepoints before and after surgery were compared across experimental groups. The vertical and horizontal dotted lines represent an absolute fold change of 0.5 and a p-value of 0.05 respectively
