# Supplementary figures and images for: Effects of Extracorporeal Shockwave Therapy on Functional Recovery and Circulating miR-375 and miR-382-5p after Subacute and Chronic Spinal Cord Contusion Injury in Rats
Source: Biomedicines. 2022 Jul 7;10(7):1630. doi: 10.3390/biomedicines10071630 (PMC9313454; doi:10.3390/biomedicines10071630)

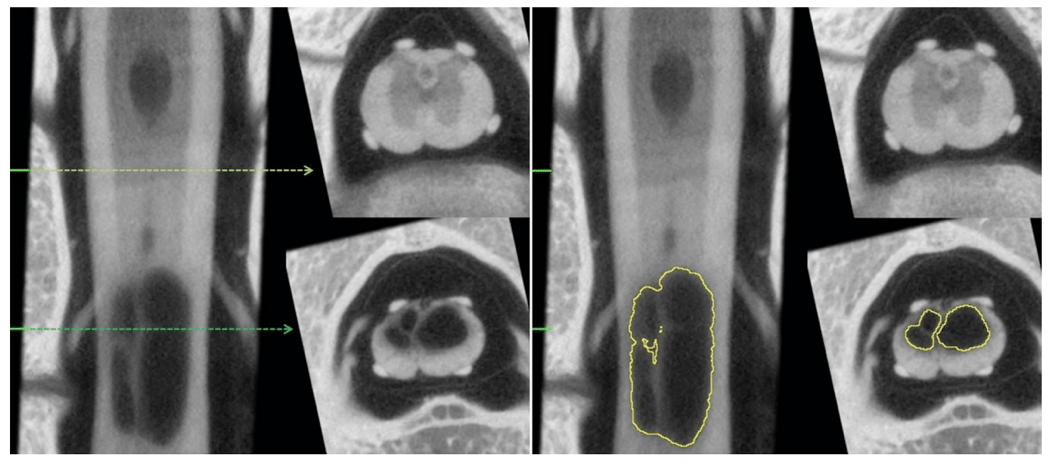

Supplement: Supplementary file 1 [file biomedicines-10-01630-s001.zip › Supplementary figure S1.tif]

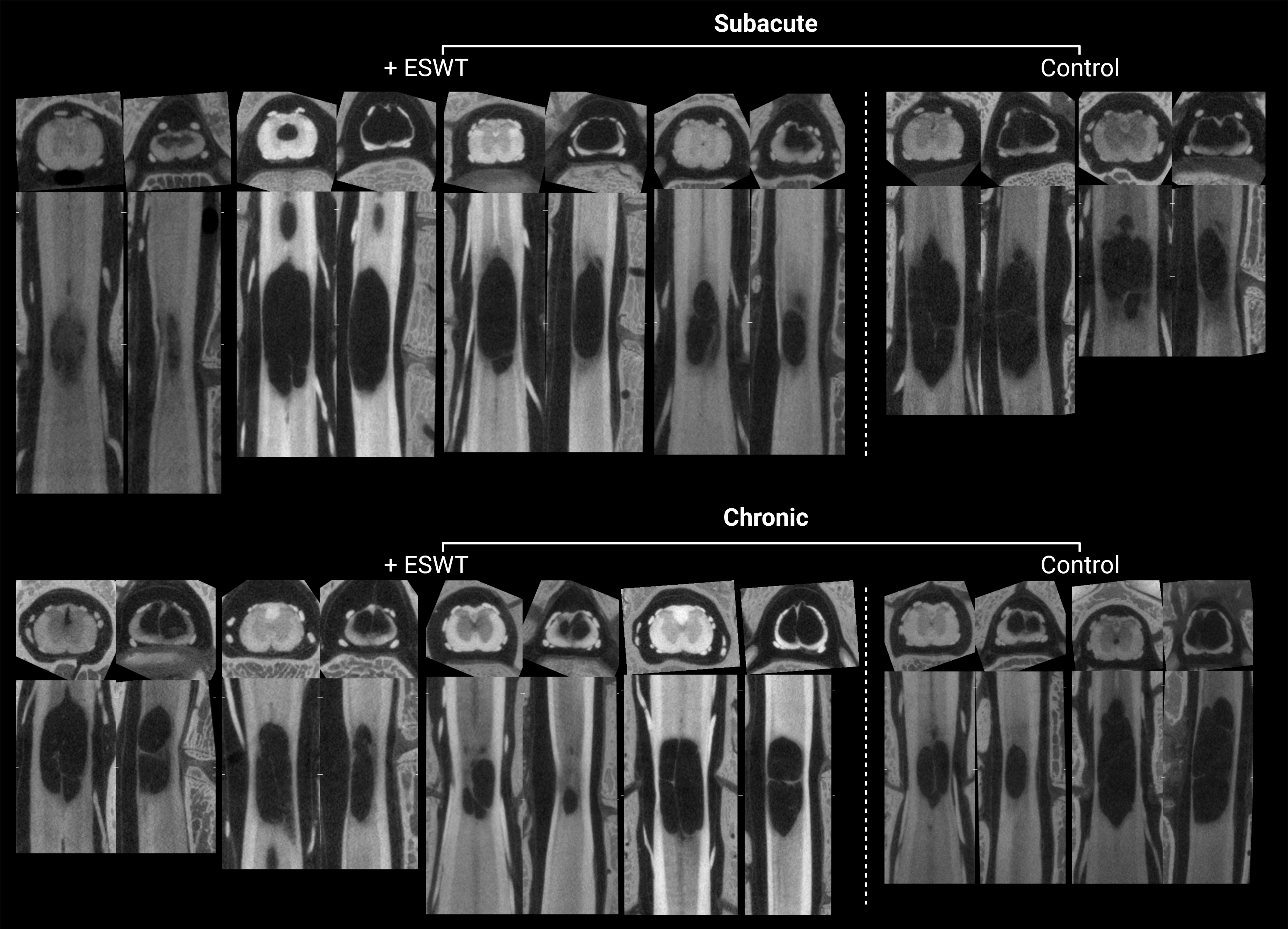

Supplement: Supplementary file 1 [file biomedicines-10-01630-s001.zip › supplementary figure S2.png]

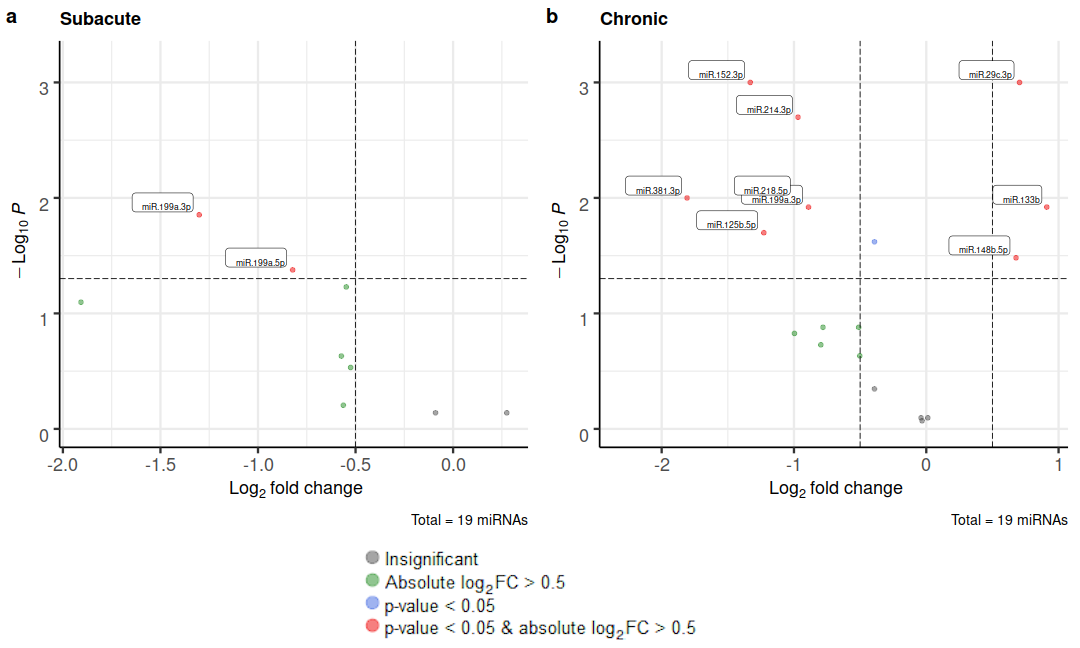

Supplement: Supplementary file 1 [file biomedicines-10-01630-s001.zip › Supplementary Figure S3.png]
